# Current knowledge of targeted-release budesonide in immunoglobulin A nephropathy: A comprehensive review

**DOI:** 10.3389/fimmu.2022.926517

**Published:** 2023-01-04

**Authors:** Jian Liao, Yijing Zhou, Xiuqin Xu, Ke Huang, Pengtao Chen, Yuhao Wu, Biao Jin, Qianlong Hu, Guanlin Chen, Shankun Zhao

**Affiliations:** ^1^ Department of Nephrology, Jiaxing Hospital of Traditional Chinese Medicine, Jiaxing, Zhejiang, China; ^2^ Department of Clinical Medical School, Taizhou University, Taizhou, Zhejiang, China; ^3^ Department of Urology, Taizhou Central Hospital (Taizhou University Hospital), Taizhou, Zhejiang, China

**Keywords:** IgA nephropathy, budesonide, targeted-release formulation, renal function, proteinuria

## Abstract

Immunoglobulin A (IgA) nephropathy is a common autoimmune kidney disease. Accumulating studies showed that IgA nephropathy may be partially correlated with mucosal immune system dysfunction. Systemic corticosteroid treatment exerts an essential protective effect against renal deterioration in IgA nephropathy. However, long-term use of corticosteroids may cause systemic side effects. The novel targeted-release formulation (TRF) of budesonide has been shown to deliver the drug to the distal ileum with the aim of minimizing adverse events for patients with IgA nephropathy. In this review, we have summarized all the current evidence of the effects of TRF-budesonide protecting against IgA nephropathy. Three randomized controlled trials (RCTs), one cohort, two case reports, and an ongoing Phase 3 trial (Part B, NCT03643965), were under comprehensive review. These included studies demonstrated that TRF-budesonide could remarkably reduce proteinuria, hematuria, and creatinine, as well as preserve renal function. The local immunosuppressive effects exhibited by TRF-budesonide may represent a novel and promising approach to treating IgA nephropathy. However, the current evidence was only derived from limited trials. Therefore, more well-designed RCTs are still warranted to validate the curable profile of TRF-budesonide in treating IgA nephropathy.

## Introduction

Immunoglobulin A (IgA) nephropathy is an autoimmune kidney disease, characterized by the deposition in the glomerular mesangial region with mesangial cell proliferation. IgA nephropathy was first described by Jean Berger in 1968 (1), and now it has become one of the most common primary glomerulonephritis worldwide (2, 3). In Europe, the prevalence of IgA nephropathy was reported to range from 19 to 51% of renal biopsies (4). IgA nephropathy is one of the important causes of primary glomerulonephritis. It is more common in children than adults and has various clinical manifestations, about 20%-40% of IgA nephropathy patients will progress to end-stage renal disease (ESRD) within 20 years after diagnosis (5). Based on the data from the genome-wide association studies (GWAS), East Asian origin people are more liable to develop IgA nephropathy than those African-descendant individuals (6). The confirmation of IgA nephropathy can be detected by the renal biopsy with the anomalous deposition of IgA in the glomerular mesangium (7). However, some of the patients with IgA nephropathy are clinically silent cases, thus the biopsy may not properly diagnose this disease. The prevalence of IgA nephropathy varies greatly across different countries due to various socioeconomic factors, genetic susceptibility, renal biopsy, and urine analysis (8). Hypertension, persistent proteinuria (especially persistent proteinuria of >1g/day), cigarette smoking, and decreased glomerular filtration rate (GFR) are the major risk factors for progression to ESRD (9, 10).

Angiotensin-converting enzyme inhibitors (ACEIs) or angiotensin receptor blockers (ARBs) are the first-line treatment for IgA nephropathy (11). Kidney Disease Improving Global Outcomes (KDIGO) guidelines recommend that all patients with proteinuria of >0.5 g/day be treated with ACEIs or ARBs, regardless of whether they have hypertension (recommendation level 1B), and suggest up-titrating ACEIs or ARBs to a maximum recommended dose or maximum tolerated dose to reduce proteinuria to <0.75-1 g/day (recommendation level 1B) (12). Systemic corticosteroid therapy can be considered for 6 months if optimized renin-angiotensin system (RAS) blockade with persistent proteinuria is more than 0.75-1 g/day after 3 months. In children, treatment with corticosteroids could significantly decrease mesangial proliferation. Nahoko Yata et al. (13) reported that children previously treated with corticosteroids may have a renal survival in 10 years after the end of the treatment. However, systemic corticosteroids should be used with caution or avoided in estimated glomerular filtration rate (eGFR) <50 mL/min/1.73m^2^, obesity, osteoporosis, and other conditions (recommendation level 2B) (12). However, the recent STOP-IgA nephropathy trial (14) and the TESTING trial (15) showed that although immunosuppressive (IS) and systemic corticosteroids can effectively reduce proteinuria, the incidence of adverse events is high (mainly infection). Therefore, the use of IS for the treatment of primary IgA nephropathy in patients with a high risk of progression remains controversial.

## Pathogenesis of IgA nephropathy

### Roles of Gd-IgA1 in IgAN

IgA is predominantly produced in the bone marrow and is the primary immunoglobulin at mucosal surfaces, where it plays a crucial role to protect the host from antigens, it can be structurally divided into IgA1 and IgA2 subclass (16). The main difference between IgA1 and IgA2 is that the hinge region of IgA1 contains 19 amino acids and 6 potential *O*-lined glycans, However, IgA2 has only 10 amino acids and no *O*-lined glycans, this structural difference makes IgA1 sensitive to bacterial proteases (17). However, abnormalities in the process of galactosylation of IgA1 will lead to the formation of poorly *O*-galactosylated IgA1 (also referred to as galactose-deficient IgA1, or Gd-IgA1), which can induce the production of anti-Gd-IgA1 antibodies, this is considered to be the initiating factor for IgA nephropathy (18). At the present stage, the internationally recognized theoretical basis of IgA nephropathy disease formation is the four-hit hypothesis: serum Gd-IgA1 is obviously elevated in circulation (16). Gd-IgA1 induces the production of autoantibodies. Antibodies with specificity for Gd-IgA1 (anti-Gd-IgA1 antibodies) are elevated and form Gd-IgA1 containing circulating immune complexes (Gd-IgA1 CICs) with Gd-IgA1, those antigen-antibody complexes widely deposit in the glomerular mesangial region. Glomerular mesangial region deposition of Gd-IgA1 CICs can trigger local inflammation, leading to mesangial cells releasing cytokine and the complement system being activated, which can result in glomerular injury (19).

Gut mucosal immune system and mucosal-derived Gd-IgA1 are considered to involve in the pathogenesis of primary IgAN. Peyer’s patches are collections of lymphoid follicles found in the mucosal layer of the intestine, and concentrated in the ileum (20). They are belonging to gut-associated lymphoid system, functioning as antigen sampling and inductive sites and being a source of mucosal B cells that express Gd-IgA1 (21). High levels of Gd-IgA1 in the circulation are observed in patients with IgAN, forming immune complexes with IgG and IgA autoantibodies. As these immune complexes accumulate in the mesangium, inflammatory and fibrotic cascades develop, causing progressive kidney damage (22). On the other hand, Gd-IgA1 CICs with anti-Gd-IgA1 antibodies deposited in the glomerular mesangial region may induce mesangial cell proliferation and extracellular matrix production, leading to cytokine production, which disrupts podocyte function, damaging the glomerular barrier and tubulointerstitial (5, 23). Since Gd-IgA1 plays an important role in the pathogenesis of IgAN, the drug targeting the gut-associated lymphoid system might reduce Gd-IgA1 production by inhibiting mucosal B-lymphocyte activation and Peyer plaque proliferation and effectively treat the disease (24). The pathogenesis of IgA nephropathy provides a theoretical basis for the locally targeted treatment of mucosal immune system disorders.

It has been established that the mucosal immune system is involved in the pathogenesis of primary IgA nephropathy, in which mucosal B lymphocytes located in Peyer plaques produce Gd-IgA1 in response to microbial or dietary antigens. which The mucosal immune system can form Gd-IgA1 CICs with anti-Gd-IgA1 antibodies in circulation and then deposit in the glomerular mesangial region, triggering local inflammation. resulting in This action may induce mesangial cells cell proliferation and extracellular matrix production, leading to cytokine production, which disrupts podocyte function, compromising damaging the glomerular barrier and tubulointerstitial (5, 22). The pathogenesis of IgA nephropathy provides a theoretical basis for the locally targeted treatment of mucosal immune system disorders. Therefore, reducing Gd-IgA1 production by inhibiting mucosal B-lymphocyte activation and Peyer plaque proliferation can also become a new way to treat primary IgA nephropathy (23).

### Roles of Fc alpha R1 in IgAN

IgA1-sCD89 may play a major role in IgAN pathogenesis. IgA1 can bind to the IgA Fc receptor (CD89/FcαRI), expressed by myeloid cells, inducing the release of soluble CD89 and creating IgA-sCD89 immunocomplexes (25). CD89 is a glycoprotein found primarily on the surface of myeloid cells, where it acts as an Fc receptor for IgA. CD89 is a glycoprotein found primarily on the surface of myeloid cells, where it acts as an Fc receptor for IgA IgA binding can induce CD89 shedding, producing a soluble form of the receptor (sCD89) which is postulated to complex with gd-IgA1 to form one of the four possible gd-IgA1 CICs (26). In IgA nephropathy, IgA1 binds and promotes CD89 shedding from circulating myeloid cells, leading to circulating IgA1–sCD89 complexes. The complexes could be accumulated in the mesangium, thus stimulating the production of the chemokines as well as the cytokines. Besides, inflammation could be also promoted, therefore causing a renal injury of IgA nephropathy (27). FcαRI is an IgA receptor found in immune response cells of the myeloid lineage. In IgAN patients, the expression of FcαRI in monocytes is restricted to almost only the full-length FcαRI, a transmembrane arrangement. Therefore, the extracellular domain of this receptor can suffer proteolytic cleavage, leading to the formation of the GdIgA1-FcαRI immune complex (28). In IgAN patients, the expression of FcαRI in monocytes is restricted to almost only the full-length FcαRI, a transmembrane arrangement. Therefore, the extracellular domain of this receptor can suffer proteolytic cleavage, resulting in the formation of the GdIgA1-FcαRI immune complex.

### Roles of microbiodata in the progression of the IgAN

It is now well established that the interaction between IgA and the microbiota promotes homeostasis with the host to prevent disease. The gut-kidney axis may play a key role in the development of IgAN. Human IgAN was suggested to be a maladaptive host response to the microbiota. Moreover, intestinal microbiota and its metabolites play a key role in IgA-immune responses. The function of the microbiota and mucosal immunity in the development of IgAN rests centrally. Advancement in the field of knowledge on the role played by gut microbiota exposure in patients susceptible to developing IgAN was proposed by genome-wide association studies (GWAS) (29). The gut microbiota is known to be implicated in the host’s innate and adaptive immune system. The composition of the gut flora is based on the intestinal immune system that protect against pathogens through the production of IgA. Microbial infections can stimulate the differentiation of B-cells into IgA-secreting plasma cells. In the development of IgAN, an aberrant mucosal immune response to commensal microbiota, which is triggered by the hyperactivation of IgA-promoting cytokines. The intestinal microbiota contributes to the progression of IgAN in a spontaneous murine strain producing high levels of IgA (30).

Since microbiota dysbiosis was found to be associated with the incidence and progression of IgAN (31), antibiotics could be used to treat or prevent IgAN development or progression. Moreover, gut microbiota manipulation might be a new option for the therapy of IgAN, including dietary interventions, prebiotics, and probiotics, or through fecal microbiota transplantation.

The use of oral prednisone was found to improve renal function in patients with IgAN (32). It was reported that ACE inhibitors assuredly reduce blood pressure and proteinuria, being associated with better renal survival (33). Thus, the Kidney Disease: Improving Global Outcomes (KDIGO) guidelines for glomerulonephritis recommend ACE inhibitors as first-line treatment for patients with IgA nephropathy with proteinuria of more than 1 g/day (recommendation level 1B) (34). According to this theory, some investigators found that corticosteroids plus ACE-inhibitors provided additional benefits compared with ACE-inhibitors alone in preventing the progression of IgAN in long-term follow-up (35).

### Other mechanisms for IgAN

There is a lot of recently published data on the origin of galactose-deficient IgA1 molecules and some other potential etiopathological components of IgA. IgA nephropathy was found to be associated with gastrointestinal tract infections, indicating dysregulation of mucosal immune response might be involved in the development of IgAN. In IgAN patients, B lymphocytes from Peyer’s patches are primed to produce Gd-IgA1 in response to microbial or dietary antigens and this is proposed to be the earliest pathogenic event. Gd-IgA1 binds to glomerular mesangial cells resulting in stimulation of cell proliferation, the release of inflammatory mediators that promote proteinuria, and fibrotic remodeling, ultimately leading to loss of renal function. A recent study (24) showed that surface/membrane bound-Gd-IgA1 positive cells expressed dominantly L chains of the lambda isotype in the serum of the IgAN patients. In addition, Gd-IgA1 positive cells are favored in homing to digestive tracts due to the expression of cellular receptors. A high level of IgA1 with lambda L chains in the plasmablasts/plasma cells of the IgAN patients might be a response to the action of the immune system. Thus, abundant migratory Gd-IgA1 lambda positive cells predestined for homing to digestive tract mucosal tissues might be one of the key pathological factors for IgAN development. These novel findings may contribute to the development of causative therapy for IgAN by targeting the Gd-IgA1 lambda isotype during digestive tract infections.

Aberrantly glycosylated IgA plays an important role in the pathogenesis of IgAN. Recently, Kano et al. (36) demonstrated that nasal-associated lymphoid tissue (NALT) might be the prominent production site for aberrantly glycosylated IgA. The pathogenesis of IgAN might be correlated to the activation of the TLR9-mediated pathway. The authors found that aberrant glycosylation of IgA leading to IC formation through overactivation of TLR9 in the NALT is an essential event in IgAN development. This result indicated that targeting overexpression of TLR9 in the NALT might be a promising therapeutic approach for IgAN.

The above studies demonstrate that it is an effective therapeutic approach toward targeting specific genes for treating IgAN through the digestive tract.

### Targeted-release budesonide in IgA nephropathy

It was found that there is a positive relationship between inflammatory bowel disease (IBD) and the risk of IgA nephropathy (37), suggesting the immune mechanisms might play roles in the development of IgA nephropathy. The immunopathogenesis of IgA nephropathy may be associated with the dysfunction of gut-associated lymphoid tissues (38). These correlations indicated the possible benefits of budesonide, a drug targeting intestinal immunity and local inflammation in the gut mucosa, for treating IgA nephropathy. Targeted Release Formulation (TRF) of Budesonide (Nefecon^®^) is modified by TARGIT starch capsule technology to deliver a local potent anti-inflammatory effect in the distal ileum and proximal colon, where Peyer plaque density is highest (39, 40). TRF budesonide thus acts on the local immune hyperresponsiveness. After absorption, there are approximately 70% of the active compounds of TRF-budesonide to be released in the distal ileum and proximal colon. they are first metabolized in the liver by the cytochrome P450 isoenzyme CYP3A4/CYP3A5 to produce compounds with low glucocorticoid activity (16α-hydroxyprednisone and 6-β -hydroxybudesonide), ultimately less than 10% Active compounds enter systemic circulation Thus, although the glucocorticoid effects of budesonide targeted release agents are stronger than those of prednisone, the low systemic bioavailability offers greater safety (39–41).

TRF-budesonide as a new treatment method has been used in IgA nephropathy, in high-risk IgA nephropathy patients can effectively reduce hematuria and proteinuria, keep stable renal function, fewer side effects, and high security (42). TRF-budesonide in the treatment of IgA nephropathy has attracted increasing attention, although the KDIGO guidelines for its use have not been clearly laid out (12). In this article, we aim to explore whether the currently available literature supports recommending the use of TRF-budesonide for the treatment of IgA nephropathy.

## Litereture search

To maximally detect the eligible studies that met the theme of TRF-budesonide in treating IgA nephropathy, we have conducted a systematic review of the four common-used databases, including MEDLINE, EMBASE, Google Scholar, and the Cochrane Library. The keywords for searching the potential articles in MEDLINE were: ((((((((((((((“Glomerulonephritis, IGA”[Mesh]) OR (Glomerulonephritides, IGA)) OR (Berger’s Disease)) OR (Bergers Disease)) OR (IGA Glomerulonephritis)) OR (Nephropathy, IGA)) OR (Iga Nephropathy 1)) OR (Nephropathy 1, Iga)) OR (Immunoglobulin A Nephropathy)) OR (Nephropathy, Immunoglobulin A)) OR (Nephritis, IGA Type)) OR (IGA Type Nephritis)) OR (Berger Disease)) OR (IGA Nephropathy)) AND [(Targeted Release Formulation of Budesonide) OR (nrf of budesonide)]. Six studies, including three RCTs (20, 42, 43), one cohort (44), and two case reports (45, 46) were finally included for further review. A specific data collection table was applied to extract the main data from each study, e.g. the first author’s name, publication year, study design, the sample size of the study and/or placebo group, the administration of TRF of Budesonide, the main findings within the study, and the adverse events of TRF of Budesonide. **Table 1** showed the summary of the eligible studies reporting TRF of Budesonide against IgA nephropathy.

**Table 1:** Characteristics and the main findings in the six included studies.

Study	Study design	Sample size of the Study group	Sample size of the placebo group	Administration of TRF of Budesonide	Main findings	Adverse events
Smerud et al. (42) (2011) Sweden	RCT	16	0	TRF-Budesonide (Nefecon^®^) 8 mg/day was given orally to the 16 patients for 6 months, followed by a 3-month follow-up.	The median reduction in urine albumin (U-albumin) was 529 mg/day (relative reduction of 23% from baseline) during the treatment period (P=0.04), and after a follow-up of 2 months the median reduction in U-albumin a peaked at 40%. The relative reduction in serum creatinine of 6% from baseline (median: 105 μmol/L) after treatment (P=0.003).	Abdominal pain (12.5%)
FellstrÖm et al. (43) (2017) Sweden	RCT	16 mg/day TRF-budesonde (Nefecon^®^) group: 50 8 mg/day TRF-budesonde (Nefecon^®^) group: 50	50	TRF-budesonde (Nefecon^®^) was given orally 1h before breakfast for 9 months, 3-month follow-up period.	At 9 months the relative reduction of TRF-budesonide (16 mg/day plus 8 mg/day) in mean UPCR of 24.4% from baseline (change in UPCR vs placebo 0.74, P=0.0066). At 9 months, mean UPCR had decreased by 27.3% in the patients who received TRF-budesonide 16 mg/day (P=0.0092) and 21.5% in the patients who received TRF-budesonide 8 mg/day (P=0.0290), the patients who received placebo had increased by 2.7%. During the treatment, eGFR remained stable in the TRF-budesonide groups but decreased in the placebo group, with a mean percentage change from baseline of 9.8% at 9 months in the placebo group, 0.6% at 16 mg/day group, 0.9% at 8 mg/day group (16 mg/day vs placebo 1.12%, P=0.0026; 8 mg/day vs placebo 1.10%, P=0.0064). The effect was sustained throughout follow-up and incidence of adverse events was similar in all groups.	Nasopharyngitis (20%^a^, 16%^b^, 20%^c^) Acne (18%^a^, 16%^b^, 6%^c^) Joint swelling (18%^a^, 16%^b^, 4%^c^) Cushingoid (16%^a^, 10%^b^, 6%^c^) Insomnia (16%^a^, 12%^b^, 4%^c^) Diarrhoea (10%^a^, 2%^b^, 14%^c^) Dyspepsia (14%^a^, 4%^b^, 8%^c^) Headache (12%^a^, 6%^b^, 6%^c^) Alopecia (8%^a^, 8%^b^, 4%^c^) Back pain(6%^a^, 12%^b^, 2%^c^) Mood swings (10%^a^, 6%^b^, 4%^c^) Oedema peripheral (12%^a^, 4%^b^, 4%^c^) Blood creatine phosphokinase increased (6%^a^, 6%^b^, 6%^c^) Hirsutism (10%^a^, 6%^b^, 2%^c^) Hypertension (10%^a^, 6%^b^, 2%^c^) Muscle spasms (4%^a^, 10%^b^, 4%^c^) Abdominal pain(6%^a^, 8%^b^, 2%^c^) Nausea (6%^a^, 8%^b^, 2%^c^) Upper respiratory tract Infection (6%^a^, 4%^b^, 6%^c^)
Ismail et al. (44) (2020) Romania	Retrospective cohort	Budesonide (Budenofalk) group: 18	Systemic steroids group: 18	TRF-budesonide (Budenofalk) 9mg/day was given orally for 12 months, then reducted to 3mg/d for 12 months.	At 24 months, the renal function of TRF-budesonide group decline of - 0.22ml/min/1.73m^2^, compared to -5.89 ml/min/1.73m^2^ in the corticosteroid treatment group (P=0.44). The median reduction in proteinuria was 45% in the TRF-budesonide group and 11% in the corticosteroid group at 24 months (P=0.009). Throughout the treatment period, TRF-budesonide was well tolerated with minimal side effects.	Nausea (6%), Viral upper respiratory tract infection (6%) Oral candidiasis (6%)
Venettacci et al. (45) (2018) Australia	Case- report	a 12-year-old boy	NA	during this 6-month trial, TRF-Budesonide 3 mg/day was given orally for 4 weeks,then increased to 6 mg daily.	His UPCR reduced to 69 mg/mmol and serum creatinine falled to 69 μmol/L, and no side effects were reported.	NA
Lingaraj et al. (46) (2020) India	Case- report	a 34-year-old male patient	NA	TRF-budesonide 9 mg/day was given orally for 9 months, then reduced to 3 mg/d and continued.	After 6 months of treatment, the serum creatinine of the patient was 1.1 mg/dL and urine tests were normal, and no side effects were reported during the treatment.	NA
Barratt et al. (20) (2022) UK	RCT phase 3	97 patients (over 18 Years)	102	Nefecon 16 mg once daily by mouth for 9 months	After 9 months, UPCR was 27% lower in the Nefecon group compared with placebo (*P*=0.0003). A statistically significant 3.87 ml/min per 1.73 m2 eGFR treatment benefit (*P*= 0.0014) for Nefecon was observed. The improvement in 1-year eGFR slope was 3.37 ml/min per 1.73 m2/yr (P= 0.0111) with Nefecon compared with placebo.	Most TEAEs (e.g. hypertension, peripheral oedema, muscle spasms, and acne) were reversible and categorized as mild or moderate in severity.

^a^16 mg/day TRF-budesonde (Nefecon^®^) group, ^b^8 mg/day TRF-budesonde (Nefecon^®^) group, ^c^placebo capsules group. UPCR, urinary protein-to-creatinine ratio; NA, not available.

## Discussion

### The main findings reported in he included studies

Mounting studies have confirmed the excellent effects of steroids on IgAN. A recent international, multicenter, and double-blind RCT (47) demonstrated that oral methylprednisolone for 6 to 9 months dramatically improved the renal function or alleviated kidney failure in patients with IgAN. A previous meta-analysis (48) included 58 relevant studies revealed that corticosteroid treatment probably improved clinical outcomes in adults and children with IgA nephropathy and proteinuria. A lately published meta-analysis (49) reported that corticosteroids combined with immunosuppressants might significantly reduce the risk of proteinuria and end-stage kidney disease in IgAN patients. However, adverse events under oral steroids treatment should be acknowledged, especially in those with high-dose therapy. To reduce systematic adverse effects under oral steroids treatment, novel drugs delivering the glucocorticoid locally with limited systemic exposure has been designed.

Smerud et al. first used TRF-budesonide in the treatment of IgA nephropathy and published their research results in 2011 (42). All patients had a proteinuria (urine albumin) of >500 mg/day (median: 1579 mg/day) despite optimized RAS blockade or treatment with immunosuppressive or systemic corticosteroid agents, then they administrated TRF-budesonide 8 mg/day to the 16 patients for 6 months, followed by a 3-month follow-up. The median reduction in urine albumin (U-albumin) was 529 mg/day (relative reduction of 23% from baseline) during the treatment period (P=0.04) and after a follow-up of 2 months. The median reduction in U-albumin peaked at 40%. The relative reduction in serum creatinine of 6% from baseline (median: 105 μmol/L) after treatment (P=0.003). The serum HAA-IgA levels (a measure of serum Gal-deficient IgA1) were no significant differences between before and after treatment. Two patients withdrew prematurely from this trial due to adverse events related to abdominal pain, and no glucocorticoid-related side effects were observed. The polymeric IgA1 in glomerular mesangial of IgA nephropathy is mainly derived from the mucosal immune system, therefore, identifying and eliminating the gastrointestinal antigens would be a new therapeutic strategy in IgA nephropathy. Based on this theory, Smerud et al. (42) applied TRF-budesonide to patients with IgA nephropathy and achieved exciting results, this exploratory trial demonstrated the use of TRF-budesonide in the management of IgA nephropathy can effectively reduce proteinuria and improve renal function, with good safety. Although this trial had some limitations which the treatment period of only 6 months and no control group, the results provide a new therapeutic strategy for the treatment of IgA nephropathy.

Based on the results of the exploratory phase 2a trial (42), FellstrOm et al. did a randomized, double-blind, placebo-controlled phase 2b trial (NEFIGAN) (43), they aimed to evaluate the safety and efficacy of two different doses of TRF-budesonide in patients with IgA nephropathy who were at risk of progression to end-stage renal disease due to persistent proteinuria despite optimized RAS blockade therapy. This study included a 6-month run-in, 9-month treatment, and 3-month follow-up (43). Eligible adult patients with persistent urine protein creatinine ratio (UPCR) ≥0.5 g/g (or persistent proteinuria ≥0.75 g/day) and eGFR (or GFR) ≥45 mL/min/1.73m^2^ despite 6 months’ optimized RAS blockade (6-month run-in phase) were enrolled into the 9-month treatment phase. After screening, 150 eligible adult patients with primary IgA nephropathy were assigned 1:1:1 to TRF-budesonide 16 mg/day, TRF-budesonide 8 mg/day, and placebo, respectively. All patients continued to optimize RAS blockade therapy by up-titrating ACEIs or ARBs (or both) as far as tolerated to the maximum recommended dose throughout this trial. After treatments with 9 months, the relative reduction of TRF-budesonide (16 mg/day plus 8 mg/day) in mean UPCR of 24.4% from baseline (change in UPCR vs placebo 0.74, P=0.0066). Meanwhile, mean UPCR had decreased by 27.3% in the patients who received TRF-budesonide 16 mg/day (P=0.0092) and 21.5% in the patients who received TRF-budesonide 8 mg/day (P=0.0290), the patients who received placebo had increased by 2.7%. During the treatment, eGFR remained stable in the TRF-budesonide groups but decreased in the placebo group, with a mean percentage change from baseline of 9.8% at 9 months in the placebo group, 0.6% at 16 mg/day group, 0.9% at 8 mg/day group (16 mg/day vs placebo 1.12%, P=0.0026; 8 mg/day vs placebo 1.10%, P=0.0064). The effect was sustained throughout follow-up and the incidence of adverse events was similar in all groups. This NEFIGAN trial shows that TRF-budesonide can effectively delay the progression of IgA nephropathy, and dose-dependent, TRF-budesonide 16 mg/day, added to optimize RAS blockade, can more effectively reduce proteinuria and stable renal function, reduce the risk of progression to ESRD, and good security. Those results indirectly support that mucosal immune system dysfunction plays an important role in the pathogenesis of IgA nephropathy.

Subsequently, Ismail et al. (44) conducted a retrospective propensity-matched comparative trial to evaluate the efficacy of TRF-budesonide in the treatment of IgA nephropathy. Unlike the exploratory phase 2a trial (42) and Phase 2b trial (43), this trial was the first to compare TRF-budesonide with systemic corticosteroids. Eighteen of 143 IgA nephropathy patients were identified who received TRF-budesonide 9mg/d during the first 12 months and reduced to 3mg/d after 12 months. The control group were treated with systemic corticosteroids. At 24 months after treatment, the renal function of the TRF-budesonide group declined to - 0.22ml/min/1.73m^2^, compared to -5.89 ml/min/1.73m^2^ in the corticosteroid treatment group (P=0.44). The median reduction in proteinuria was 45% in the TRF-budesonide group and 11% in the corticosteroid group at 24 months (P=0.009). Compared with the NEFIGAN trial (40), this study provided the longest duration of TRF-budesonide treatment, further demonstrating the efficacy and safety of budesonide over systemic corticosteroids in reducing proteinuria and maintaining kidney function. A little disappointed, this was a retrospective study, and lack of evaluation of IgA1 and Gd-IgA1 CICs before and after treatment.

In 2018, Venettacci et al. (45) reported a successful case of TRF-budesonide in the management of IgA Nephropathy in a child, an 8-year-old boy was diagnosed with IgA nephropathy and then treated with systemic corticosteroids and ACEIs/ARBs, however, his serum creatinine increased to 90 μmol/L and UPCR to 520 mg/mmol. At 12 years of age, he stopped taking systemic corticosteroids and commenced a six-month trial of TRF-budesonide, he received TRF-budesonide at 3 mg/d and increased to 6 mg/d after 4 weeks. Finally, his UPCR was reduced to 69 mg/mmol, and serum creatinine fell to 69 μmol/L, and no side effects were reported. Although this was a single case, this successful case was the first to use TRF-budesonide in a child with IgA nephropathy. This case suggests that TRF-budesonide may play a role in the treatment of IgA nephropathy in children, providing a new therapeutic direction for children with IgA nephropathy. In 2021, Lingaraj et al. (46) reported a 34-year-old male patient who was a recipient of a living-related renal allograft, 2 years after transplantation, this patient presented with lower limb swelling, serum creatinine increased from 1.1 mg/dL to 2.5 mg/dL, 24-hour proteinuria quantification was 3g, and renal biopsy results suggested IgA nephropathy, then he was treated with TRF-budesonide 9 mg/d except for triple-drug immunosuppressive therapy. After 6 months of treatment, the serum creatinine of the patient was 1.1 mg/dL and urine tests were normal, and no side effects were reported during the treatment. At present, recurrent glomerulonephritis is increasingly considered as the main cause of late renal transplantation dysfunction (50), and long-term oral immunosuppression in such patients brings greater challenges to the treatment of recurrence. This case showed that TRF-budesonide for IgA nephropathy after kidney transplantation seems to be a promising treatment, but needs to be confirmed and evaluated the long-term effect on allograft function by more multi-center studies.

At present, the global phase 3 clinical trial (NeflgArd trial) is currently underway to evaluate the efficacy and safety of TRF-budesonide (Nefecon^®^) 16mg/d versus placebo in adult patients with primary IgA nephropathy who are receiving optimal RAS blockade and are at risk of progressing to ESRD, 24-hour proteinuria quantification of the enrolled patients was >1 g/d or UPCR >800 mg/mmol, and eGFR was 35-90 ml/min/1.73m^2^ (https://clinicaltrials.gov/, ClinicalTrials.gov Identifier: NCT03643965). The first Part of the trial (Part A) is just finished. The results of the phase 3 NefigArd trial (20) demonstrated that the urine protein-to-creatinine ratio (UPCR) was 27% lower in the Nefecon group (16 mg/d, orally for 9 months) compared with placebo, along with a benefit in eGFR preservation corresponding to a 3.87 ml/min/1.73 m2 difference versus placebo (all *P*<0.05), which confirmed the findings from the phase 2b NEFIGAN study. For safety assessment, most TEAEs (e.g. hypertension, peripheral edema, muscle spasms, and acne) were reversible and categorized as mild or moderate in severity. Discontinuations from treatment due to TEAEs were low (9.3% in the Nefecon and 1.0% in the placebo groups). The NeflgArd trial is still being a 12-month followed-up to observe changes in eGFR after drug withdrawal to evaluate the long-term effects of Nefecon on delaying the progression of IgA nephropathy. In addition to the exciting Part A results, we are looking forward to the results of the whole global phase 3 clinical trial.

Treatment of primary IgA nephropathy is based on supportive therapy, the latest 2021 KIDGO guidelines recommend that all patients with proteinuria of >0.5 g/day should be treated with ACEIs or ARBs (a maximum recommended dose or a maximum tolerated dose), regardless of whether they have hypertension (recommendation level 1B) (12). Systemic corticosteroid therapy can be considered for 6 months if optimized renin-angiotensin system (RAS) blockade with persistent proteinuria is more than 0.75-1 g/day after 3 months(recommendation level 2B) (12). The evidence for IS in IgA nephropathy is insufficient. The recent STOP-IgA nephropathy trial (14) and the TESTING trial (15) showed that although IS and systemic corticosteroids can effectively reduce proteinuria, the incidence of adverse events is high (mainly infection). In the current trials, the application of TRF-budesonide in the treatment of IgA nephropathy can effectively reduce proteinuria and delay renal function progression with good safety, providing a new option for the treatment of IgA nephropathy. On December 15, 2021, the United States Food and Drug Administration (FDA) approved TRF-budesonide (Nefecon/TarpeyoTM) for the treatment of primary IgA nephropathy in adults, it became the first targeted drug approved for IgA nephropathy. We look forward to the results of the NeflgArd trail and the subsequent global phase 4 clinical trial to further verify the efficacy and safety of TRF budesonide and provide a more reliable evidence-based medical basis for the treatment of IgA nephropathy.

### The systemic effects and adverse events under budesonide treatment

According to the published data, the most common adverse effects of budesonide are hypertension, peripheral edema, muscle spasms, acne, dermatitis, weight gain, dyspnea, facial edema, dyspepsia, fatigue, and excess hair. As reported by Fellström et al, the incidence of adverse events (i.e., nasopharyngitis, acne, joint swelling, and gastrointestinal complaints, etc) with TRF-budesonide was broadly similar to that seen with the placebo (43). The retrospective study (44) conducted by Ismail et al. showed that 4/18 (22%) of the patients in the budesonide treatment group encountered adverse events, including mild gastrointestinal complaints (nausea), mild oral candidiasis, mild episode of upper respiratory tract infection, and deep vein thrombosis complicated with pulmonary embolism. However, thrombosis event was considered unrelated to budesonide treatment due to this diagnosis was prior to IgAN diagnosis. In the study (42) developed in Sweden, the investigators found three out of sixteen (3/16) of the patients had adverse events (i.e., abdominal pain, sleep disturbances, and increased micturition) under TRF-budesonide therapy. The recent multi-center, double-blind, randomized, placebo-controlled NefIgArd trial (20) demonstrated that TRF-budesonide treatment was well-tolerated, and treatment-emergent adverse events were mostly mild to moderate in severity and reversible. Based on the above evidence, similar to oral steroids treatment, TRF-budesonide therapy related adverse events seems unavoidable, but these side effects are reversible.

### Comparisons betweenNefecon^®^ and Budenofalk^®^

Different from Nefecon^®^, some formulations of the locally acting glucocorticoid budesonide were also developed. Some of them are approved for the treatment of mild to moderate active IBD. For example, Budenofalk is a gastro-resistant, pH-modified formulation of budesonide with a maximum release of active compound in the distal ileum and proximal colon (44). Additionally, it has an extensive first-pass metabolism and induces only mild and transient reductions in plasma cortisol levels, thus sparing steroid-related side effects. After absorption, budesonide undergoes extensive first-pass metabolism *via* cytochrome P450 isoenzymes CYP3A4/CYP3A5 in the liver, with only about 10% of active compound finally entering systemic circulation (41). Thus, despite a stronger glucocorticoid effect than prednisone, the low systemic bioavailability offers the advantage of a safer profile.

TRF-budesonide, also named Nefecon, is a novel, oral, targeted-release formulation of the glucocorticosteroid budesonide (43). Nefecon was developed to release the drug in the distal ileum, which has a high density of Peyer’s patches. It was reported that mucosal immune system dysfunction has a significant role in the pathogenesis of IgAN. TRF-budesonide can target the region of the gastrointestinal tract where Peyer’s patches reside at high density. Local immunosuppression of mucosal B-lymphocyte activation and proliferation in Peyer’s patches could attenuate GdIgA1 production. Nefecon was designed as a targeted-release oral dosage form of Budesonide, which is subject to high first-pass metabolism, resulting in low systemic exposure.

The principal difference between Budenofalk and Nefecon is the latter is designed for targeted-release formulation of budesonide *via* targeting intestinal mucosal immunity (where Peyer’s patches reside at high density) upstream of IgAN manifestation, while the former acts on the whole digestive tract with a maximum release of budesonide in the distal ileum and proximal colon. The strength of both Budenofalk and Nefecon significantly reduces corticosteroid-related adverse events. A prospective interventional open-label single-center non-randomized Phase IV study aimed to evaluate the efficacy and safety of Budenofalk in treating IgAN is ongoing. The hypothesis of this study is that Budenofalk for the treatment of IgAN offers the premise of a safer approach than systemic corticosteroids by targeting GALT dysregulation with a pH-modified formulation of budesonide with a maximum release in the distal ileum and proximal colon.

## Conclusion

Based on the current evidence, patients with IgA nephropathy could remarkably gain clinical benefit from TRF budesonide treatment, featured by declination of proteinuria and the stableness of renal function. In addition, TRF budesonide is well tolerated, with minimal adverse events. As a result, this local immunosuppressive treatment may represent a novel and promising approach to treating IgA nephropathy. However, only one relevant multicenter RCT is ongoing. Thus, more well-designed RCTs are still warranted to validate the curable profile of TRF budesonide for treating IgA nephropathy. On the other hand, nanomedicine has excellent potential in combining different drugs to deliver them to the digestive tract. Therefore, future RCTs are recommended to design as a combination therapy of targeted-release formulation of budesonide with ACE inhibitors and the drugs of gut microbiota manipulation (i.e., antibiotics, prebiotics, and probiotics) for treating IgAN.

## Author contributions

All authors listed have made a substantial, direct, and intellectual contribution to the work and approved it for publication.
